# Chemoenzymatic synthesis

**DOI:** 10.1038/s42004-025-01451-z

**Published:** 2025-03-13

**Authors:** Paweł Borowiecki, Sandy Schmidt

**Affiliations:** 1 Laboratory of Biocatalysis and Biotransformation, Department of Drugs Technology and Biotechnology, Faculty of Chemistry, Warsaw University of Technology, Warsaw, Poland; 2 Department of Chemical and Pharmaceutical Biology, Groningen Research Institute of Pharmacy, University of Groningen, Groningen, The Netherlands

## Abstract

*Communications Chemistry* is pleased to introduce a Collection of research works focused on recent developments within the interdisciplinary field of chemoenzymatic synthesis. Here, the Guest Editors highlight key themes and look towards the future of this research field.

In the last few decades, biocatalytic transformations using naturally occurring (native) and/or engineered enzymes as environmentally friendly catalysts with excellent chemo-, regio-, and stereoselectivity have emerged as a versatile and highly sustainable synthetic tool^1^. In this context, biocatalytic methods offer mild reaction conditions (i.e., ambient temperature and pressure, neutral pH, aqueous reaction media, etc.) and superb atom economy with low-level waste and by-product formation^2,3^. Thus, biocatalytic strategies can be used as an alternative to traditional catalytic strategies for manufacturing high-value-added organic molecules, including pharmaceuticals, agrochemicals, flavors, fragrances, food additives, cosmetics, natural products^2–7^, etc. Consequently, the use of biocatalytic strategies in synthesis design can often overcome several drawbacks, mainly related to the high cost of organo- and transition-metal catalysts, harsh reaction conditions (i.e., elevated temperatures, high pressures, use of flammable and explosive gases), and environmental hazards resulting from the excessive use of volatile organic solvents and highly toxic metals^8^. In addition, the incorporation of enzymes into chemical processes often simplifies a particular technology by shortening the synthetic route, eliminating the need for protecting group strategies, and using costly and highly specialized chemical equipment^9^.

Moreover, various engineering concepts, including protein engineering, biocatalyst formulation (i.e., immobilization), media engineering, and/or reactor engineering, have moved biocatalysis as a scientific field from the ‘purely academic avant-garde’ to an industrially attractive technology useful for innovative manufacturing processes^10^. Furthermore, novel chemoenzymatic concepts to increase the level of efficiency, sustainability, and applicability of biocatalysis, such as the development of multi-enzymatic cascades^11–13^, chemoenzymatic cascades^14–18^, and photo-biocatalytic cascades^19–24^, as well as multifunctional biocatalysts^25,26^, enzyme promiscuity^27–30^, multi-enzyme fusion systems^31–35^, microbial cell factories^36–39^, and nature-inspired enzyme-based artificial metabolic pathways consisting of complex biocatalytic reaction networks (so-called ‘systems biocatalysis’)^40–43^, have greatly expanded the application of enzymes in organic synthesis. Noteworthy are also novel enzymes capable of catalyzing selective C–X (where X stands for C, O, N, S, Si, etc.) bond formation^44–49^, selective oxidation^50–54^ and/or reduction reactions^55–57^, complex multicomponent reactions^58–60^, cleavage of challenging chemical bonds such as Si–C^61,62^, or depolymerization of polymeric products to their precursors or raw materials^63–66^, all of which have led to the successful overcoming of long-standing synthetic challenges in organic chemistry.

In this collection, we are presenting a series of innovative studies that highlight significant and fascinating advances as well as challenges in the field of biocatalysis. These contributions cover three main topics: (1) enzyme discovery and engineering, (2) chemoenzymatic reactions, and (3) chemoenzymatic total synthesis.

## Enzyme discovery and engineering

Implementing biocatalytic transformations in an industrial setting is often determined by the enzyme performance required to achieve the productivity needed to make such a process industrially viable^67^. In addition, the portfolio of chemical transformations accessible to biocatalysis is challenged by the sheer number of chemical reactions available to other catalytic strategies^68^. In this context, the discovery of novel enzymes and protein engineering strategies that allow the improvement of catalytic activity, selectivity, specificity, and operational stability of biocatalysts have become invaluable tools for expanding the repertoire of transformations currently possible with enzymes. This collection includes several examples highlighting how the discovery and characterization of novel biocatalysts and their engineering via computational or (semi)-rational design significantly expands the scope of accessible transformations while improving the robustness under process-related conditions.

Zhang et al. developed a rapid protocol for Increasing-Molecule-Volume Screening to identify three imine reductases (IREDs) with a preference for bulky amine substrates (10.1038/s42004-022-00743-y)^69^. Increasing the scope of IREDs is of particular interest because of their potential application in the asymmetric synthesis of chiral amines. However, the industrial application of IREDs is mainly limited by their small substrate range and typically low activities towards bulky amines. Interestingly, the identified IR-G02 IRED has a wide substrate range and has been used to synthesize over 135 secondary and tertiary amines with significant conversions. A gram-scale synthesis of an analog of the active pharmaceutical ingredient (API) cinacalcet was carried out using a kinetic resolution approach with high enantioselectivity (>99% *ee*) and 48% conversion.

Another intriguing example highlighting the power of protein engineering to develop improved biocatalysts for the synthesis of APIs is demonstrated by Malca et al., who optimized a ketoreductase (KR) from *Sporidiobolus salmonicolor* for the chemoenzymatic synthesis of ipatasertib, a potent protein kinase B inhibitor (10.1038/s42004-024-01130-5)^70^. By combining mutational scanning and structure-guided rational design, a variant with ten amino acid substitutions exhibited a 64-fold higher apparent *k*_cat_ and improved robustness under process conditions compared to the wild-type enzyme. Moreover, machine learning-aided enzyme engineering led to the design of smaller-size libraries for screening. The identified best KR variant was implemented in the final biocatalytic process, yielding the alcohol intermediate with ≥98% conversion and a diastereomeric excess of 99.7% (*R*,*R*-*trans*) from 100 g L^−1^ ketone after 30 h.

The α-oxoamine synthases (AOSs) are a class of pyridoxal 5’-phosphate (PLP)-dependent, irreversible, carbon-carbon bond-forming enzymes, which have been limited previously by their narrow substrate specificity and requirement of acyl-CoA thioester substrates. Campopiano et al. reported the structure-guided engineering of ThAOS to arrive at variants able to use a greatly expanded range of amino acids and simplified *N*-acetylcysteamine (SNAc) acyl-thioester substrate (10.1038/s42004-025-01448-8s)^71^.

Another important aspect of the industrial applicability of biocatalytic approaches is the enzyme’s thermostability, which enhances its utility under the harsh conditions of the final process. In this regard, Go et al. employed a computational design strategy to improve both the thermostability and enzymatic activity of the diterpene glycosyltransferase UGT76G1, an enzyme critical for the industrial production of steviol glucosides, which are natural sweet-tasting compounds (10.1038/s42004-023-01070-6)^72^. By combining stabilizing mutation scanning with a Rosetta-based protein design protocol, the obtained variant exhibited a 9 °C increase in apparent *T*_m_, a 2.5-fold increase in product yield, and a significant reduction in by-product formation. This work demonstrates that computer-assisted structure-based design can be a very powerful tool for improving not only the thermostability of an enzyme but also its overall catalytic properties.

Asparaginyl ligases have been extensively utilized as valuable tools for site-specific bioconjugation or surface modification. However, the application is hindered by the laborious and poorly reproducible preparation processes, unstable activity and ambiguous substrate requirements. Tang et al. employed a structure-based rational approach to engineer a recombinant asparaginyl ligase with appreciable catalytic activity across a wide pH range. This enhancement in catalytic efficiency provides valuable insights into site-specific modifications using efficient recognition and nucleophile motifs (10.1038/s42004-024-01173-8)^73^.

Another strategy for obtaining biocatalysts with enhanced properties is ancestral sequence reconstruction (ASR). This sequence-based protein design method predicts ancestral sequences from a multiple sequence alignment and phylogenetic tree^74,75^. Interestingly, ancestral enzymes often exhibit improved properties, such as enhanced substrate selectivity, increased thermostability, and better soluble expression. Thus, ancestral sequences often serve as a superior starting point for protein engineering aimed at enhancing desired properties. For example, Kawamura et al. used ASR to design a novel l-amino acid oxidase (HTAncLAAO2), which already demonstrated high thermostability and long-term stability (10.1038/s42004-023-01005-1)^76^. After structure-guided mutagenesis of HTAncLAAO2 to improve its activity towards l-tryptophan, the variant W220A was developed, exhibiting a more than 6-fold increase in *k*_cat_ for l-Trp compared to the wild type. This variant serves as a promising starting point for the design of novel oxidases with high activity towards a broad range of amines and amino acids.

Eggerichs et al. reported a streamlined approach for rationally selecting enzymes with desired functionalities from the ever-increasing amount of available sequence databases. By studying the sequence-function relationships, they demonstrated this approach using 4-phenol oxidoreductases as a case study (10.1038/s42004-024-01207-1)^77^. Eight enzymes from the oxidase branch were selected out of 292 sequences based on the properties of the first-shell residues within the catalytic pocket, using the computational tool A^2^CA as a guide.

Modular polyketide synthases (PKSs) play a vital role in the biosynthesis of complex natural products with pharmaceutically relevant properties. Buyachuihan et al. demonstrated that the promiscuous malonyl/acetyl-transferase domain (MAT) from murine fatty acid synthase is a highly versatile tool for producing polyketide analogs. They showcased MAT-based reprogramming of polyketide biosynthesis through domain swapping as a straightforward strategy for the regioselective modification of substituents decorating the polyketide scaffold (10.1038/s42004-024-01269-1)^78^.

Computational methods are crucial tools for studying enzymatic mechanisms and guiding enzyme engineering. Zhang et al. used quantum chemical calculations to investigate the kinetic resolution of α-methyl-phenylacetaldehyde by norcoclaurine synthase, shedding light on the mechanism of stereoselectivity exhibited by this enzyme in catalyzing the Pictet-Spengler reaction of dopamine with chiral aldehydes (10.1038/s42004-024-01146-x)^79^. Ju et al. used quantum chemical calculations to investigate the effects of olefin substitutes, non-native amino acid axial ligands, and natural and non-natural macrocycles with the widely used ethyl diazoacetate. Their study aims to aid in the design of efficient heme-inspired biocatalysts for the carbene transfer reactions with olefines, leading to the production of value-added cyclopropanes (https://www.nature.com/articles/s42004-024-01371-4)^80^.

## Chemoenzymatic reactions

The combination of multiple catalytic steps involving enzymes to achieve highly precise chemical transformations has provided a wide range of prime examples of how biocatalysis enhances the efficiency, sustainability, and applicability of organic synthesis for the production of diverse high-value compounds^13,81,82^. Such cascading reactions can integrate a diverse panel of catalytic concepts, ranging from homogeneous, heterogeneous, organo-, and photocatalysis, and can be performed either simultaneously or sequentially^14,83^. The combination of different catalytic systems not only reduces the effort and waste associated with intermittent processing but can also facilitate thermodynamically challenging transformations or avoid unstable or toxic intermediates. This collection features several compelling examples that illustrate how single and multi-step enzymatic transformations, as well as their combination with chemocatalytic steps, greatly expand the application potential of enzymes in organic synthesis.

In this context, Petermeier et al. demonstrated an efficient chemoenzymatic process involving the enzyme-catalyzed decarboxylation of bio-based phenolic acids in wet cyclopentyl methyl ether (CPME), followed by a base-catalyzed acylation to produce various acetylated hydroxystyrenes (10.1038/s42004-024-01138-x)^84^. With the goal of developing a productive, safe, and low-waste process, the authors identified and optimized several critical aspects of the entire cascade, with a particular focus on the enzymatic step. The key to success was the continuous control of the water activity during the reaction. By incorporating a water reservoir, the process was intensified, ultimately leading to a sequential two-step cascade capable of operating at high substrate loads (400 g L^−1^) and achieving complete conversion in less than 3 hours.

Another intriguing example demonstrating the synergy of chemo- and enzymatic catalysis in accessing high-value-added compounds is the work reported by Peh et al. (10.1038/s42004-023-01083-1)^85^ In this study, a novel flavin-dependent halogenase was characterized, with its activity and specificity explored for both its native substrate and a range of xenobiotic pyrroles. To demonstrate the applicability and scalability of PrnC-catalyzed biocatalytic halogenation, the authors used the enzyme in the chemoenzymatic synthesis of a C-3 chlorinated analog of the agrochemical fungicide fludioxonil. The chemoenzymatic approach involved a palladium-mediated Suzuki coupling to construct the respective biaryl pyrrole precursor, followed by late-stage, site-selective chlorination catalyzed by PrnC, yielding the fungicide (fludioxonil) analog in a 58% isolated yield.

While these examples illustrate that combining chemical and enzymatic reaction steps is a facile approach that can be efficiently executed in concurrent or sequential reaction modes, the use of purely enzymatic transformations can also be highly effective in producing synthetically valuable fluorinated chemicals. In this context, Nieto-Domínguez et al. reported an in vitro enzymatic cascade towards mono- and trifluorinated alanine enantiomers (10.1038/s42004-024-01188-1)^86^. The authors combined an alanine dehydrogenase and a diaminopimelate dehydrogenase with a formate dehydrogenase-based cofactor regeneration system for the in vitro stereocomplementary production of both enantiomers of 3-fluoroalanine. The designed cascade proved efficient in obtaining optical isomers of the titled fluorinated amino acid in >85% yield.

A wide variety of pharmaceuticals can be conveniently synthesized using chemoenzymatic methods, particularly with high stereoselectivity. In this context, Dander et al. developed a one-pot sequential chemoenzymatic method for converting amides into enantiomerically enriched alcohols. This approach combines a nickel-catalyzed Suzuki-Miyaura coupling of amides in aqueous medium with an asymmetric biocatalytic reduction, furnishing a wide range of diarylmethanol derivatives in high yields and enantiomeric excess (up to 99%) (10.1038/s42004-019-0182-8)^87^. Moreover, the formal syntheses of both enantiomers of the pharmaceutical orphenadrine were accomplished using stereocomplementary ketoreductases (KREDs), highlighting the synthetic utility of this chemoenzymatic transformation. In turn, Farkas et al. reported the synthesis of *trans*-4-substituted cyclohexane-1-amines, including a key precursor to cariprazine, through a transaminase-catalyzed stereoselective deamination of their diasteromeric mixtures and/or diastereotope selective amination of the corresponding ketone (10.1038/s42004-024-01148-9)^88^. Rudzka et al. reported an asymmetric transfer hydrogenation of prochiral carbonyl derivatives using a variant of an alcohol dehydrogenase deduced from *Lactobacillus kefir*, producing (*R*)-alcohols of high pharmaceutical relevance (10.1038/s42004-023-01013-1)^89^.

Bicyclic peptides exhibit improved metabolic stability and target specificity compared to their linear or mono-cyclic counterparts. However, their synthesis remains challenging due to their intricate architectures. Kobayashi et al. presented a highly selective and operationally simple one-pot chemoenzymatic tandem cyclization approach for synthesizing bicyclic peptides with small to medium ring sizes, utilizing thioesterase-mediated head-to-tail cyclization followed by copper(I)-catalyzed azide-alkyne cyclization (10.1038/s42004-024-01147-w)^90^.

Despite the increasing demand for efficient and sustainable chemical processes, developing scalable systems for fine chemical production using biocatalysis remains a significant challenge. As a proof of concept, Lim et al. (10.1038/s42004-024-01288-y)^91^ developed a scalable flow system using immobilized enzymes to facilitate flavin-dependent biocatalysis. This system integrates a flavin-dependent Old Yellow Enzyme (OYE) with a soluble hydrogenase to enable H_2_-driven regeneration of the OYE cofactor FMNH_2_, targeting asymmetric reduction of various cyclic enones. The interdisciplinary development of an integrated process concept, converting glucose to 4,5-dimethyl-1,3-dioxolane via 2,3-butanediol, demonstrates the full potential of diverse catalytic systems through a sequential tailor-made combination of microbes, enzymes and chemo-catalysts (10.1038/s42004-023-01052-8)^92^.

## Chemoenzymatic total synthesis

Natural products are typically complex molecules containing multiple stereocenters in their structures, implying the formation of numerous regio- and stereo-isomers, which are naturally produced in vivo through the action of enzymes as the key components of cellular metabolism. Therefore, it is evident that enzymes, or whole cells containing them, represent one of the most powerful catalytic tools for obtaining these compounds in vitro with excellent yields and under precise stereochemical control.

This collection highlights several creative efforts to develop efficient biocatalytic systems for natural product synthesis. For instance, Milzarek et al. showcased the streamlined formation of structurally complex sorbicillinoids (10.1038/s42004-023-00996-1)^93^. In this context, the first total synthesis of spirosorbicillinols A–C was achieved by combining enzyme-catalyzed synthesis of sorbicillinol, using the heterologously overexpressed monooxygenase SorbC, with the chemical synthesis of various scytolide analogs. Moreover, this article demonstrates the significant potential of chemoenzymatic approaches in the ten-step synthesis of the shikimic acid-derived natural product scytolide, along with a series of different double bond/C-8 stereoisomers. The reported total syntheses are elegant examples of how innovative bioorganic chemistry can enable synthetic access to the natural product family of the spirosorbicillinols, as well as several unnatural diastereomers. Another article by Müller et al. (10.1038/s42004-024-01126-1)^94^ presents clear advantages for developing innovative synthetic routes to sorbicillactone A and its unnatural C9 analogs, which exhibit potent anti-leukemic and anti-HIV activities. The reported asymmetric synthesis of these molecules proved to be more straightforward and efficient compared to established synthetic routes, which only yielded racemic nitrogen-containing sorbicillinoids. Notably, the key steps of this highly convergent, stereoselective, and concise route were the enantioselective oxidative dearomatization of sorbillin to sorbicillinol, catalyzed by the enzyme SorbC, followed by a Michael addition using a fumaryl azlactone building block.

The paper by Jin et al. reports on a highly modular chemoenzymatic cascade assembly (MOCECA) strategy for the tailor-made, large-scale synthesis of ganglioside analogs with diverse glycan and ceramide epitopes (10.1038/s42004-024-01102-9)^95^. This innovative method enabled the synthesis of structurally well-defined gangliosides on an industrially relevant (heterogram) scale. The availability of these precisely defined structures allowed the authors to compare them with synthetic analogs, resolving the issue of conflicting descriptions for GM1 components found in different pharmaceutical documents (i.e., GM1 patent applications and drug package inserts) by accurately reinterpreting the two-component structures of the commercialized GM1 drug named Sygen™. The catalytic modules of the developed MOCECA strategy were characterized by simplicity, high yield, and excellent compatibility, making them a potentially powerful synthetic tool for designing and synthesizing new glycolipid drugs targeting central nervous system diseases.

Nguyen et al. discovered two cytochrome P450 monooxygenases derived from *Camptotheca acuminata* that catalyze regio-specific 10- and 11-oxidations of camptothecin (10.1038/s42004-021-00602-2)^96^. The authors demonstrated that combinatorial chemoenzymatic C-H functionalizations of the camptothecin scaffold can produce key precursors for anticancer drugs, including topotecan (Hycamtin^®^) and irinotecan (Camptosar^®^). This innovative work is expected to pave the way for further regioselective functionalization of rigid polycyclic alkaloid structures, leading to the development of new bioactive molecules.

Seibel et al. reported a targeted β-amino acid-specific homology-based multi-query search to identify potential bacterial macrolactam producers (10.1038/s42004-023-01034-w)^97^. Based on in-depth genomic and metabolomic analyses, the authors validated two ciromicin A producers from the genus *Amycolatopsis* sp. It is envisioned that combining targeted mining with network-based analysis will facilitate the discovery of previously unreported naturally occurring macrolactams.

Skellam et al., in their review article, discussed various strategies for the combinatorial biosynthesis of natural products through the engineering of fungal enzymes and biosynthetic pathways (10.1038/s42004-024-01172-9)^98^. They also presented examples of new-to-nature bioactive molecules synthesized using combinations of fungal and non-fungal enzymes. These efforts are expected to advance the remarkable and complex chemistry involved in the total synthesis of natural products and inspire further biosynthetic campaigns focused on developing their semi-synthetic analogs.

## Outlook

In the near future, we expect to see more significant efforts to develop new biocatalysts and chemoenzymatic strategies to tackle catalytic reactions that are currently considered too complex and, therefore, less appealing to the chemical industry. Significant untapped potential remains in artificial cascade processes that mimic the metabolism of living organisms by combining mulitiple enzymes in a single reaction vessel to obtain complex molecules without the need to isolate intermediates and with the possibility of overcoming unfavorable thermodynamics. In this field, complementary advantages and attractive features of other sustainable methodologies directly derived from photo- and/or electro-chemistry should motivate researchers to elaborate novel visible-light-driven and/or electrochemically-driven enzyme-catalyzed transformations. Moreover, protein fusion strategies aimed at simplifying specific reactions or enhancing cofactor recycling systems will become one of the essential pillars of modern biocatalysis. Currently, we are also witnessing a surge in ‘unnatural biocatalysis’, which enables a wide range of challenging stereoselective C-C and C-heteroatom bond-forming transformations through the directed evolution of enzymes. In addition, significant progress has been made in developing multifunctional biocatalysts capable of transforming different functional groups within a single substrate molecule, making them suitable even for the late-stage functionalization of fine chemicals or natural products. An impending ‘wave of biocatalysis’ is expected to create novel opportunities for advancing cutting-edge technologies, enabling the cost-effective production of chiral APIs and functional materials. Given the urgent need to reduce the carbon footprint of industrial processes and minimize waste disposal on our planet, we hope to see significant progress in biofuel production, energy supply and storage, as well as the controlled recycling and biodegradation of synthetic polymers, such as polyethylene terephthalate (PET) plastics, and other persistent pollutants. Therefore, the continued development of engineering methodologies, supported by computational tools such as artificial intelligence (AI) based on artificial neural networks and machine learning (ML) algorithms, is highly desirable to unlock novel enzyme functionalities and fully harness their catalytic potential for practical and industrial applications.
